# Diagnosis and treatment evaluation in patients with spontaneous intracranial hypotension

**DOI:** 10.3389/fneur.2023.1145949

**Published:** 2023-03-10

**Authors:** Dwij Mehta, Sanjay Cheema, Indran Davagnanam, Manjit Matharu

**Affiliations:** ^1^Headache and Facial Pain Group, University College London (UCL) Queen Square Institute of Neurology, The National Hospital for Neurology and Neurosurgery, London, United Kingdom; ^2^Lysholm Department of Neuroradiology, UCL Queen Square Institute of Neurology and National Hospital for Neurology and Neurosurgery, London, United Kingdom

**Keywords:** spontaneous intracranial hypotension, CSF hypovolaemia, CSF hypovolemia, CSF leak, orthostatic headache, CSF venous fistula

## Abstract

Spontaneous intracranial hypotension is characterized by an orthostatic headache and audiovestibular symptoms alongside a myriad of other non-specific symptoms. It is caused by an unregulated loss of cerebrospinal fluid at the spinal level. Indirect features of CSF leaks are seen on brain imaging as signs of intracranial hypotension and/or CSF hypovolaemia as well as a low opening pressure on lumbar puncture. Direct evidence of CSF leaks can frequently, but not invariably, be observed on spinal imaging. The condition is frequently misdiagnosed due to its vague symptoms and a lack of awareness of the condition amongst the non-neurological specialities. There is also a distinct lack of consensus on which of the many investigative and treatment options available to use when managing suspected CSF leaks. The aim of this article is to review the current literature on spontaneous intracranial hypotension and its clinical presentation, preferred investigation modalities, and most efficacious treatment options. By doing so, we hope to provide a framework on how to approach a patient with suspected spontaneous intracranial hypotension and help minimize diagnostic and treatment delays in order to improve clinical outcomes.

## Introduction

Spontaneous intracranial hypotension (SIH) is a disorder that presents with a varied clinical presentation and is caused by leakage of cerebrospinal fluid (CSF) at the level of the spine. Broadly, three types of CSF leaks are recognized: ventral leak from dural tear secondary to calcified disc herniation or an osteophyte, leaking meningeal diverticula, and CSF-venous fistula (CVF).

The estimated incidence of SIH is 5 per 100,000 (1), although this is likely to be an underestimation due to its varied clinical presentation in conjunction with suboptimal diagnostic criteria and investigative methodology. SIH is more prevalent in women with a female to male ratio of approximately 2:1. Mean age at presentation is around 42 years of age (2). There is a possible association with genetic connective tissue disorders such as hypermobile Ehlers-Danlos (hEDS) and Marfan syndrome; however, there is mixed evidence in prospective studies and requires further investigation (3, 4).

Orthostatic headache is often, although sometimes erroneously, deemed synonymous with a diagnosis of SIH. A recent systematic review by D'Antona et al. showed prevalence of headaches in 97% of patients with SIH, and 92% of these had an orthostatic headache. Non-orthostatic headaches are also well recognized in SIH and a diagnosis of SIH cannot be excluded clinically on the basis of a non-orthostatic headache (2).

Beyond headaches, numerous associated symptoms can occur and include nausea, vomiting, neck discomfort, and audiovestibular symptoms. More serious complications include cerebral venous sinus thrombosis, bibrachial amyotrophy, and superficial siderosis (5). Behavioral variant frontotemporal dementia-like syndrome in SIH is also now increasingly recognized and has been termed as frontotemporal brain sagging syndrome (FBSS) or brain sagging dementia (BSD) (6, 7).

For diagnosis of SIH, the International Classification of Headache Disorders (ICHD-3) requires either low CSF opening pressure on lumbar puncture of <6 cm H_2_O or imaging features of CSF leakage on imaging in absence of a procedure or trauma that can result in CSF leakage (Table 1). As discussed later in the article, there is a need to refine this as current criteria can lead to underdiagnosis of the condition.

**Table 1:** International Classification of Headache Disorders 3rd edition diagnostic criteria for headaches associated with low CSF pressure and spontaneous intracranial hypotension.

**Headache attributed to low CSF pressure**	**Headache attributed to SIH**
A. Any headache fulfilling criterion C.	A. Headache fulfilling criteria for *Headache attributed to low CSF pressure*, and criterion C below.
B. Either or both of the following: 1. Low CSF pressure (<60 mm CSF) 2. Evidence of CSF leakage on imaging	B. Absence of a procedure or trauma known to be able to cause CSF leakage.
C. Headache has developed in temporal relation to the low CSF pressure or CSF leakage or led to its discovery.	C. Headache has developed in temporal relation to occurrence of low CSF pressure or CSF leakage or has led to its discovery.
D. Not better accounted for by another ICHD-3 diagnosis.	D. Not better accounted for by another ICHD-3 diagnosis.

There is a lack of uniformity between centers in the diagnostic work-up and subsequent treatment options. Investigations can include neuroaxis magnetic resonance imaging (MRI) with or without contrast, magnetic resonance myelography (MRM), digital subtraction myelography (DSM), computed tomography (CT) myelography, CT skull base, radionucleotide cisternography, and lumbar puncture. Treatment options include conservative management (e.g., bed rest, analgesics, hydration, caffeine), epidural patching (autologous blood and/or fibrin), surgical repair, and endovascular procedures.

Variation in practice for diagnosing and managing patients with SIH reflects the deficiencies in current diagnostic criteria, as well as the lack of evidence-based consensus guidelines (8). Diversity in the clinical specialities leading the management of CSF leaks depending on which center the patient is at also leads to the heterogeneity in care described above; neurologists, neurosurgeons, interventional radiologists, and anesthetists are frequently involved in the management of SIH patients and can have different approaches to the management of SIH. Lastly, limited resources and availability of specialist personnel at non-neurological centers can also influence the management options.

In this article, we aim to propose a clinical strategy when approaching patients with orthostatic headaches, provide an overview of the investigations used for diagnosing and localizing CSF leaks, as well as outline the effective management options. We will also aim to identify areas surrounding management of SIH that are in need for further research.

## Causes and pathophysiology

### Causes

Three causes of spinal CSF leaks are currently recognized, namely: type 1–ventral leak from dural tear secondary to calcified disc herniation or an osteophyte; type 2–leaking meningeal diverticula; and type 3–CSF-venous fistula (CVF). Although there appears to be general consensus in recognizing type 3 CSF leaks as being secondary to CSF venous fistula, there is a degree of variability in how the rest are classified (9–12). Table 2 summarizes some of these variations in classification.

**Table 2:** Spinal CSF leak classifications for spontaneous intracranial hypotension.

		**Spinal CSF leak classifications**			
**CSF leak type**		**Schievink et al**. **(****12****)**	**Häni et al**. **(****11****)**	**Farb et al**. **(****10****)**	**Dobrocky et al**. **(****9****)**
Type 1	1a	Ventral dural tears secondary to calcified disc herniation or an osteophyte. Associated with ventral extradural collection.	Ventral dural tears secondary to calcified disc herniation or an osteophyte. Associated with ventral extradural collection.	Ventral dural tears secondary to calcified disc herniation or an osteophyte. Associated with ventral extradural collection.	Ventral dural tears secondary to calcified disc herniation or an osteophyte. Associated with ventral extradural collection.
	1b	Posterolateral leak not associated with disc herniation or osteophyte related tear.	Lateral leaks with visible CSF egress frequently localizing to dural tears affecting the axilla of the nerve root. Associated with meningeal diverticula.		
Type 2	2a	SIH associated with simple meningeal diverticula (>8 mm).	Meningeal diverticula without evidence of CSF egress.	Lateral proximal nerve root sleeve leak that is unrelated to degenerative disc disease. Associated with extradural collections.	Leaking spinal nerve root diverticula.
	2b	SIH associated with complex diverticula (dural ectasia).			
Type 3		CSF venous fistula. Not associated with extradural collections.	CSF venous fistula. Not associated with extradural collections.	CSF venous fistula. Not associated with extradural collections.	CSF venous fistula. Not associated with extradural collections.
Type 4		Indeterminate.	Indeterminate.	Distal nerve root sleeve leak. Not associated with extradural collections.	N/A

Ventral dural tears secondary to degenerative disc disease is a major cause of ventral extradural CSF collections, whereas CSF venous fistulae and distal nerve root sleeve leaks are not typically associated with extradural collections. A CSF venous fistula is an aberrant connection between the intrathecal space and an adjacent epidural vein (or network of veins) resulting in excessive and unregulated drainage of CSF. CSF venous fistulae are most typically seen in the mid- and lower-thoracic regions. They are frequently associated with a nerve root sleeve diverticulum (13). Kranz et al. reported that 82% of fistulae originated from a diverticulum (14).

Schievink et al. (12) reviewed 568 consecutive patients with confirmed SIH as per ICHD criteria. They noted 26.6% of patients had type 1 CSF leaks, 42.3% had type 2 CSF leaks, and 2.5% had type 3 CSF leaks. They also noted an indeterminate group that accounted for almost 29% of the patients. In this group, no cause of CSF leak was identified and up to 50% of these patients had no evidence of extradural CSF collection or other spinal abnormality on serial imaging. It is probable that a significant proportion of these patients with no discernible cause of CSF leak are in fact CSF venous fistulas, which can be particularly difficult to diagnose. It is also worth noting that only 20% of patients with type 2 CSF leak, most common cause of SIH in this study, had a demonstrable CSF leak on spinal imaging, and thus the presence of meningeal diverticula in isolation does not necessarily prove causation (12).

### Pathophysiology

Amongst the many roles of CSF, it plays a crucial role in providing buoyancy. There is between 90 and 150 mL of CSF within the craniospinal system at any one point, with two thirds of the total volume being in the spinal compartment during recumbency. The CSF pressure along the neuroaxis is equal during recumbency. On becoming upright, the pressure within the intracranial space becomes negative, compared to a positive pressure within the thoracolumbar compartment (15). Owing to compliance of the spinal column, some extra CSF shifts into the spine and away from the intracranial compartment on becoming upright; however, this is minimal, and the intracranial pressure (ICP) is kept within the physiological range such that no orthostatic symptoms are experienced (16, 17).

Following a CSF leak there is CSF hypovolaemia and a reduction in baseline pressure. This is associated with increased compliance and thus, on becoming upright there is a greater CSF shift to the spinal compartment resulting in significant drop in ICP (17, 18). Loss of buoyancy is thought to result in less resistance to gravity causing sagging of the brain and the consequent clinical symptoms observed in SIH. Headache may be related to the traction affecting pain-sensitive nerve fibers located in the dura mater, whilst compression of the brainstem structures is thought to cause some of the other symptoms, including audiovestibular manifestations. Carlborg et al. have also previously discussed the hydromechanical hypothesis whereby changes in intracranial pressures lead to parallel changes in the perilymphatic pressure. Pathological reduction in intracranial pressure can induce endolymphatic hydrops, which clinically manifests with audiovestibular symptoms and could represent an alternative mechanism for these symptoms in SIH (19, 20). The finding of subdural haematomas is likely explained by the downward traction causing shearing of the bridging veins.

There is a significant cohort of patients who fit the criteria for SIH but have an entirely normal spine imaging with no treatment target. The underlying cause for this is poorly understood. Goldberg et al. (17) proposed a pathophysiological model based on spinal compliance where intracranial hypotension can occur in absence of a spinal CSF leak, which they referred to as “internal SIH”. The cranio-spinal CSF volume shift on becoming upright is dependent on the compliance of the spinal compartment. They suggested that presence of (1) diffuse meningeal diverticula, (2) prolapse of arachnoid through a dural tear, and/or (3) abnormally high distensibility of the dura can lead to pathologically high spinal compliance and the resultant high volume cranio-spinal shift of CSF could lead to SIH in absence of CSF leak. Proposed predisposing factors to this include (1) low total CSF volume (leading to increased compliance), (2) low intracranial CSF volume, (3) low CSF outflow resistance (CSF hypovolaemia due to increased resorption), and (4) reduced venous pressure (increased CSF resorption, CSF hypovolaemia, and intracranial hypotension) (17).

Association of connective tissue disorders such as Ehlers-Danlos syndrome and Marfan syndrome with increased risk of spontaneous intracranial hypotension may relate to pathologically increased compliance of the spinal compartment as well as an impaired structural integrity of the dura (17). Bariatric surgery has also been suggested as a risk factor for spontaneous intracranial hypotension in a case control study where 3.3% of patients with bariatric surgery had SIH, compared to 0.8% of patients with intracranial aneurysms. Proposed mechanisms include the recognized relationship between weight and intracranial pressure; obesity is linked with raised intracranial pressure as seen in idiopathic intracranial hypertension. Authors speculated that chronically elevated pressure prior to surgery may lead to weakening of the dura, which does not reverse post-operatively with the resultant susceptibility to CSF leakage (21).

Lastly, one of the chief hypotheses relating to the formation of CSF venous fistula relates to a rupture of an arachnoid granulation into an adjacent vein resulting in unregulated drainage of CSF (low CSF outflow resistance). Role of arachnoid granulations in the brain where it is involved in the drainage of CSF into the dural venous sinuses is well recognized. Studies also support the presence of arachnoid granulations along spinal nerve roots, particularly in the thoracolumbar spine, and are thought to play a role in CSF absorption in the spinal compartment (13). CVFs are most commonly found in the thoracic spine, which supports the aforementioned theory on an anatomical basis.

## Clinical approach to orthostatic headache

### Assessment

Orthostatic headache remains poorly defined and there is a lack of consensus on how fast the onset and offset of headache needs to be on becoming upright and lying down, respectively, for it to constitute an orthostatic headache. Patients with SIH frequently describe onset of headaches that varies between seconds to hours of being upright, and similarly the offset time can vary markedly. Non-orthostatic headache presentations of SIH include reverse orthostatic headache where there is paradoxical worsening with recumbency, second-half-the-day headaches, non-positional headaches, and Valsalva-induced headaches (Table 3).

**Table 3:** Approach to clinical history-taking in a patient with suspected spontaneous intracranial hypotension.

**Clinical domains**	**Clinical history**	**Notes**
Location of headache	Bilateral vs. unilateral.	Bilateral headaches are most common.
	Is it holocranial? If not, specify locations.	Diffuse/holocranial, occipital, and frontal regions are the commonest locations.
	Associated neck pain?	Neck discomfort can be seen in up to a third of patients.
Define orthostatic headache	Any headache lying down on waking up?
	Time to onset on being upright and time to maximum intensity.	Onset can be over a few hours and does not have to be immediate.
	Time to improvement in severity on lying down and time to resolution.	Offset can take up to 2 h and does not have to be immediate.
	Second-half-of-the-day headaches?	Second-half-of-the-day headaches can be missed unless specifically sought for.
	Exacerbation with Valsalva maneuvers.

Several clinicians including Mokri have made the clinical observation that some patients early in the course of the disease exhibit a clear orthostatic headache, but this component can disappear over time making the diagnosis more difficult in patients with a chronic presentation (22). Hani et al. further showed that 93% of patients presenting within 10 weeks of symptom onset in SIH had a typical orthostatic headache, compared to <63% in patients presenting after 10 weeks. This also corresponds with their observation of changing CSF fluid dynamics on lumbar infusion testing during the course of the condition; lower CSF outflow resistance and higher CSF production rates in the acute stage, which increase and decrease, respectively, in chronic state (23). It is therefore imperative to obtain a clinical history pertaining to the onset of symptoms in order to avoid missing important clues to the diagnosis.

Alongside headaches, there are a wide range of symptoms that patients with SIH can experience (Table 4) (2). It is not uncommon that the non-headache symptoms predominate and lead to marked disability. We suggest the following approach when reviewing a patient with orthostatic headache:

1) Detailed phenotyping of orthostatic headache.
2) Evaluate for associated non-headache symptoms of spontaneous intracranial hypotension.
3) Exclude secondary causes of intracranial hypotension (Table 5) and common differential diagnoses for an orthostatic headache (Table 6).
4) Consider risk factors for spontaneous intracranial hypotension (e.g., Ehlers-Danlos syndrome, Marfan syndrome, spondylosis).

**Table 4:** Non-headache presentations of spontaneous intracranial hypotension.

Nausea and/or vomiting
Extracranial pain a. Neck pain or stiffness b. Interscapular pain (can be orthostatic) c. Back pain
Audiovestibular symptoms a. Muffled hearing b. Aural fullness c. High-pitched tinnitus d. Hypoacusis e. Hyperacusis (less common) f. Vertigo g. Gait imbalance
Visual symptoms: a. Blurring b. Diplopia c. Photophobia d. Nystagmus
Cognitive symptoms: a. Subjective slowing. b. Difficulties with attention and short-term memory. c. Description of a “brain fog”. d. Behavioral variant frontotemporal dementia-like picture.
Fatigue
Orthostatic light-headedness
Facial sensory disturbance

**Table 5:** Secondary causes of intracranial hypotension.

Lumbar puncture
Spinal anesthetic (e.g., labor/cesarean section)
Spinal surgery
Other surgeries (e.g., bariatric)
Lumboperitoneal or ventriculoperitoneal shunt (leading to over-drainage)
Spinal manipulation (e.g., chiropractic)
Traumatic

**Table 6:** Differential diagnoses for orthostatic headache.

Secondary cause of intracranial hypotension (see Table 5)
Postural tachycardia syndrome
Migraine
New daily persistent headache
Craniocervical junction pathology
Colloid cyst
Idiopathic

We also inquire about the number of hours a patient spends in the day lying down. Apart from giving an indirect indication of the severity of orthostatic symptoms, it also helps highlight the degree of disability associated with it. As mentioned above, SIH affects a relatively younger population with mean age of under 43 years; the orthostatic nature of the symptoms markedly impairs patient's independence, maintain personal relationships, as well as ability to work and support themselves financially (8). We make a point of discussing patient's mental health during our reviews to identify those who may benefit from a timely psychiatric intervention.

### Differential diagnoses

Orthostatic headache is a hallmark feature of SIH and is found in over 90% of patients early in the course of the disease. Thus, there is often a tendency to erroneously surmise that all orthostatic headaches are due to SIH. There are indeed other important differential diagnoses that can also present with an orthostatic headache and must be considered when evaluating a patient with this presentation.

Migraine is an important mimic for orthostatic headaches. Patients with migraine frequently prefer to lie down in a dark room during an attack, and the improvement in headache lying still, rather than lying down, is misinterpreted as an orthostatic headache. Antecedent history of previous attacks consistent with episodic migraines with increasing frequency can help further distinguish between the two disorders.

Postural tachycardia syndrome (PoTS) can also be associated with orthostatic headaches, although accompanying symptoms of palpitations, dyspnoea, chest pain, and presyncope are important clues to the disorder (24). Patients with longstanding SIH are at risk of PoTS as a consequence of deconditioning due to prolong bed rest, which can further complicate the clinical picture when it comes to making the initial diagnosis as well as evaluating response to treatment in SIH. To minimize this potential issue, we tend to perform active stand test on all new patients with orthostatic headaches with serial monitoring.

In presence of a convincing clinical syndrome for SIH, abnormal autonomic testing does not preclude the diagnosis and necessitates further work-up for CSF leak. Working closely with the autonomic team, we would optimize management of PoTS in the first instance before consideration of invasive testing, or management, for SIH.

Hypermobility spectrum disorders, which are recognized risk factors for SIH, are also associated with a higher prevalence of both migraine and PoTS (25).

### Investigations

The diagnosis of SIH is not always straightforward as outlined above; not all orthostatic headaches are SIH, and not all SIH cases present with orthostatic headaches. Furthermore, non-headache symptoms of SIH are non-specific and many can possibly be explained by alternate headache disorders. Currently, we do not have a single investigation that can reliably and definitively exclude a diagnosis of SIH. Broadly, investigations are divided into non-invasive (e.g., MRI) and invasive (e.g., CT myelogram) imaging modalities. Alternatively, investigations can be seen as those performed to look for evidence of intracranial hypotension (e.g., brain MRI and lumbar puncture) and ones to help localize the CSF leak (myelographic imaging).

The ICHD-3 diagnostic criteria require the presence of an opening pressure that is <6 cm H_2_O on lumbar puncture, and/or presence of features on imaging that are typical for SIH. It is well established that not all patients with SIH have a low opening pressure. Studies show that only a third of patients with confirmed SIH have a pressure below 6 cm H_2_O. In fact, around 5% of patients in one study had a raised opening pressure (>20 cm H_2_O) (2, 26). In view of this, along with the invasive nature of the procedure and its potential to worsen symptoms, we avoid lumbar punctures as part of the diagnostic process. We utilize MRI of the brain and whole spine with gadolinium as our initial screening test in patients with suspected SIH. Results of the MRI are then used to carefully plan next steps in management, which would entail either a trial of non-targeted epidural blood patches, or myelographic imaging for localization of the leak to aid targeted therapy.

#### Brain MRI

**Figure 1:**
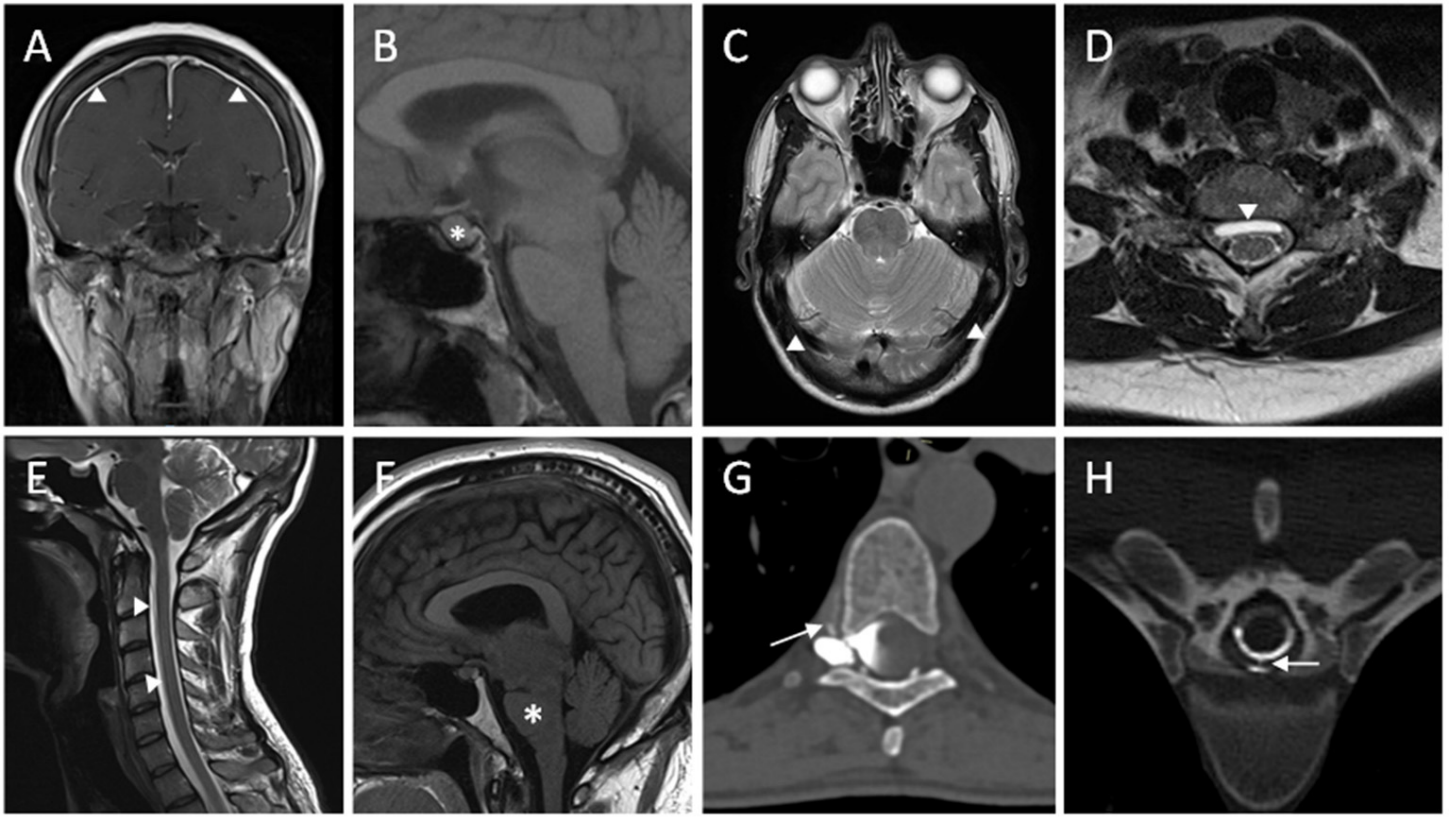
Radiological features of spontaneous intracranial hypotension. **(A)** T1W coronal post-contrast MRI sequence showing diffuse smooth pachymeningeal thickening and enhancement. **(B)** Enlargement of pituitary gland seen on T1W sagittal MRI sequence. **(C)** T2W axial MRI sequence showing distension of the transverse venous sinuses. **(D)** Ventral extradural CSF collection seen on T2W axial MRI sequence. **(E)** White arrowheads highlighting a ventral longitudinal extradural CSF collection (SLEC) in the cervical spine on T2W sagittal MRI sequence. **(F)** T1W sagittal MRI sequence demonstrating brainstem sagging. **(G)** A right-sided thoracic CSF venous fistula shown with contrast extravasation into a paraspinal vein on right decubitus CT myelography. **(H)** Extravasation of CSF through a ventral dural tear on CT myelography.

Contrast enhanced MRI of the brain is useful at highlighting features of intracranial hypotension (Figure 1). Common findings associated with spontaneous intracranial hypotension include diffuse and smooth pachymeningeal enhancement, engorged venous sinuses (“venous distension sign”), brain sagging (effacement of suprasellar and prepontine cisterns, and reduction in the mamillopontine distance), subdural collections, and pituitary enlargement. Smooth pachymeningeal enhancement is found in almost three quarters of patients and is highly suggestive of the diagnosis, although not specific to it (2, 27).

Benefits of brain MRI as a screening test include its high sensitivity, being non-invasive, free of radiation, and widely available. Despite being a sensitive test, around 20% of patients with confirmed SIH have no abnormalities (2). If brain MRI is performed very close to the onset of the symptoms, the MRI signs may not have yet developed and it may be appropriate to repeat it 1 month later if the clinical picture is suggestive (28). Thus, normal cranial imaging does not exclude the diagnosis.

Additional use of brain MRI includes the monitoring of complications associated with SIH, such as superficial siderosis, on gradient echo and susceptibility weighted imaging.

Rarely, MRI signs of SIH will be found incidentally in patients who do not have symptoms of SIH, but who have undergone MRI for another reason. As the risk of complications (such as superficial siderosis and bibrachial amotyophy) still exists in this scenario we recommend offering to investigate and treat such patients similarly to symptomatic patients. Although follow-up after treatment will be solely radiological with MRI.

#### Spinal imaging

Numerous spinal imaging modalities exist for assessment of spontaneous intracranial hypotension. The purpose of these tests is to help with the diagnosis, the localization, and determining the underlying cause of CSF leak. Spinal MRI represents a non-invasive method, whereas invasive investigations include computed tomography myelogram, digital subtraction myelography, magnetic resonance myelography (with/without intrathecal gadolinium), and radionuclide cisternography (seldom used now due to its inferior sensitivity and specificity).

We perform heavily T2-weighted, fat suppressed spinal MRI with gadolinium alongside brain MRI as part of our screening investigations. Radiological features of SIH on spinal MRI include spinal longitudinal extradural CSF collection (SLEC), dural enhancement (can be present even in absence of cranial MRI changes), and dilated epidural veins (29). MRI can also demonstrate structural abnormalities, such as presence of disc herniation, osteophytes, and nerve root cysts, which may suggest the underlying etiology.

One of the key limitations of spinal MRI is its inability to localize the site of leak given the non-dynamic nature of the scan; for example, extradural collections are particularly common at the cervicothoracic junction, which have been shown by Schievink et al. to be a false localizing sign and not necessarily reflecting the site of the leak (30). The C1-C2 sign is another example of this where there is a collection of CSF between the spinous processes of C1-2 vertebrae, and whilst a marker for CSF leak, it does not have any localizing value (31). Furthermore, extradural collections on MRI can span several vertebral levels and does not aid localization.

The primary focus of spinal MRI as part of screening is to identify patients who are positive for the presence of SLEC, which is suggestive of a high-flow leak. Majority of the SLECs are caused by a ventral dural tear and to a lesser extent by dural tears affecting the axilla of nerve root. CSF venous fistulae and distal nerve root sleeve leaks are not associated with SLEC. Thus, identification of SLEC helps with both the diagnosis of SIH and, to an extent, the underlying etiology for CSF leak. This in turn guides how the patient is positioned during subsequent myelographic imaging to maximize the yield as will be discussed below.

CT myelography involves injection of an iodinated agent into the subarachnoid space within the thecal sac in the lumbar region. Subsequent imaging of the spine as the contrast travels caudocranially allows visualization of any contrast extravasation (seen as a “split” in the contrast column) into the epidural space, and thus localizing the CSF leak (Figure 1). High-flow leaks, such as those seen with ventral dural tears, can lead to rapid extravasation of contrast agent into the epidural space following injection and by the time of image acquisition, with the contrast pooling diffusely and thereby not allowing pinpoint localization. Dynamic CT myelography has a better temporal resolution with early image acquisition from the moment contrast is being injected and is therefore helpful in localizing high-flow leaks. Identification of epidural collection beforehand on MRI also allows focusing on the area of interest when scanning. CT myelogram when compared to spinal MRI provides better structural detail and allows characterization of any calcified disc herniations and osteophytes that may have contributed to the CSF leak (32).

Digital subtraction myelography, like dynamic CT myelogram, has excellent temporal resolution that makes it useful in localization of high-volume leaks, and is utilized at many centers as an alternative to dynamic CT myelogram. Digital subtraction is also felt to improve visualization of more indistinct features associated with CSF leaks and this is particularly true in the case of CSF venous fistula.

CSF venous fistulae are a difficult entity to diagnose due to lack of extradural collections as well as the subtle findings on imaging. When managing a patient with typical clinical history and intracranial MRI findings for SIH, but negative for spinal extradural collection (SLEC-negative), CSF venous fistula should be considered with further work-up with lateral decubitus dynamic CT myelogram or digital subtraction myelography. The rationale for lateral decubitus positioning is to flood the lateral gutters within the thecal sac with contrast material and enhance visualization of the CSF venous fistula. Lateral decubitus position is also favored for nerve root sleeve leaks. For type 1 CSF leaks, which are invariably SLEC-positive, patient is positioned in the prone position (10).

Schievink et al. have looked at the detection rate of CSF venous fistula using DSM in prone vs. lateral decubitus positions in SIH patients without epidural CSF collection. A CSF venous fistula was demonstrated in 74% of the patients in lateral decubitus position, compared to 15% where DSM was performed in prone position (33). A separate study found lateral decubitus CT myelogram had a detection rate of around 50% in SIH patients without an epidural collection (34). Given the differences in patient recruitment, referral patterns, operators, and unspecified number of scans per patient to reach the diagnosis, it is not possible to directly compare these studies.

An interesting indirect sign for CSF venous fistula on CT myelogram that is receiving increasing attention is the early presence of contrast seen in the renal system, which appears to be more common in CSF venous fistula than with SLEC-positive leaks (35). This is congruent with our experience and when this sign is found in patients without an epidural collection, we carefully look for a CVF.

During CT myelogram and DSM, we also perform CSF analysis to look for early biochemical markers of superficial siderosis. These include testing for CSF cell count, ferritin, and xanthochromia.

Lastly, MR myelography with, as well as without, intrathecal gadolinium has shown promising results in investigating CSF leaks. MR myelogram without gadolinium is non-invasive and reportedly has comparable sensitivity to CT myelogram at localizing CSF leaks (36). MR myelography with intrathecal gadolinium has been shown to be effective in detecting slow-flow and intermittent leaks (32). However, utilization of intrathecal gadolinium-based approach has been limited due to its association with neurotoxicity and not being readily available.

## Treatment

There are no randomized controlled trials to guide the management of SIH. The existing evidence is based on retrospective case series and open label studies, but it suggests that most patients with SIH respond to conservative management and non-targeted epidural blood patches (EBPs). In those who do not respond, and where the site of spinal CSF can be identified using myelography, targeted treatment can be performed with patching, surgery, or transvenous embolisation.

### Conservative management

It is recognized that some spinal CSF leaks are self-limiting. Therefore, when it is first identified, patients are usually advised to undertake a short period of conservative management, hoping that the leak will heal without the need to undergo any invasive treatment.

Conservative management includes measures to both control symptoms and avoid any worsening of a CSF leak. The most common advice is bed rest and maintenance of a good level of hydration. Avoidance of any Valsalva maneuvers is also recommended, as are abdominal binders. Analgesics may be prescribed, but are often ineffective, and symptoms are primarily responsive to lying flat.

Increased oral caffeine is often recommended as it may increase intracranial pressure as an adenosine antagonist. Alternatively, it may cause a symptomatic improvement in headache due to its non-specific analgesic effect. In a double blind placebo controlled trial, oral caffeine has been shown to result in symptomatic improvement of PDPH (37). Intravenous caffeine may also be given. Theophylline, which has a similar mechanism of action, is sometimes prescribed but lacks supportive evidence. There are no controlled studies of caffeine in SIH.

The likelihood of conservative management alone being sufficient for resolution of SIH has been estimated by a meta-analysis to be 28% (95% CI 18–37%) (2). This included a heterogenous group of retrospective studies in which the proportion of responders in individual studies ranged from 3 to 55%. Opinion and practice vary on whether a patient should be recommended to undergo a minimum period of conservative management before organizing interventional treatment, or whether interventional treatment with non-targeted EBP should be performed as soon as the diagnosis is made. Our practice is to implement conservative treatments while arranging the initial non-invasive investigations but do not delay definitive treatments such as blood patches to pursue conservative treatments.

### Non-targeted epidural blood patches

The first line interventional treatment for SIH is non-targeted EBP. This involves the administration of autologous blood taken from a peripheral vein and injected into the epidural space *via* an epidural needle. EBP is typically performed in the lumbar region, due to the lower risk of spinal cord injury at this level. Aided by gravity whilst lying flat or in the Trendelenburg position, the injected blood spreads cranially in the epidural space, and therefore it can be effective independent of where in the spinal column the spinal CSF leak is located (38).

The procedure is frequently performed by anesthetists for PDPH, and by either neuroradiologists or anesthetists for SIH. The procedure is usually performed with local anesthesia, or occasionally with conscious sedation (for example, in a patient with severe needle phobia). The procedure can be performed blind by an anesthetist who is experienced in epidural anesthesia, or it can be performed using fluoroscopic or CT guidance.

The mechanism of action of EBP is unproven. Initially, the epidural blood likely causes a rise in intracranial pressure due to mass effect, often causing a short-term symptomatic improvement independent of whether the underlying CSF leak has healed. The coagulation of blood at the site of the CSF leak may then encourage a natural healing process of a dural tear, accounting for the sustained benefit from epidural blood patches (39).

A meta-analysis estimated that 64% (95% CI 56–72%) of patients have a resolution in symptoms following their first non-targeted EBP (2). The most common side effects are local back pain at the site of injection, transient paraesthesia, bruising, and post-treatment rebound headache. Nerve damage, infection and bleeding are rare complications.

Several studies have examined predictors of response to EBPs, which initially suggested that there were several radiological factors that could predict likelihood of response (40–42), but these have not been reproduced by other studies, or a recent meta-analysis (43, 44). The most important procedural predictor of response is the volume of blood injected. The cut-off for a significant difference appears to be between 20 and 22.5 ml (40, 45). Many practitioners aim for an even higher target of 40–50 ml if this is tolerated by the patient, but the injection should be halted at a lower volume if the patient experiences worsening back pain, worsening headaches, or radicular symptoms.

Non-targeted EBPs may be repeated, but with a lower response rate (41, 46–48). Once a person with SIH has not responded to two non-targeted EBPs, it is unlikely they will respond to subsequent ones; it is therefore at this point that we consider myelographic studies to identify the leak site and plan targeted treatment.

### Targeted patching

Once the site of spinal CSF leak has been located (usually with myelography), then targeted treatment can be planned. Targeted patching can be performed with blood, fibrin sealant, or a combination of the two. Open label case series have suggested possible superiority over blind autologous blood patching, although there are conflicting reports (49–52).

A retrospective evaluation comparing efficacy of fluoroscopic-guided targeted vs. blind autologous blood patch in 56 patients with SIH showed first targeted blood patch had a success rate of 87.1 vs. 52% with a blind patch (lumbar and upper thoracic regions). Current literature, as well as from authors' own experience, epidural blood patches are less efficacious when used in patients with CVF. However, Mamlouk et al. recently published a promising retrospective study where CT-guided fibrin glue injection in patients with SIH secondary to CVF resulted in excellent clinical as well as radiological outcomes (52). Further studies are required to corroborate these findings with a longer follow-up period as it can provide a minimally invasive method to managing CSF venous fistula.

Potential risks associated with targeted epidural blood patch include cord compression, intrathecal injection of the blood, and chemical meningitis along with the other risks that are associated with blind patching. Additionally, fibrin glue is associated with aseptic meningitis and arachnoiditis when administered intrathecally, and allergic reactions, including anaphylaxis. Allergic reactions are likely secondary to the presence of bovine aprotinin within the fibrin glue composition, and the risk of a reaction increases substantially on repeat exposure. There is also a theoretical risk of systemic embolisation following intravascular fibrin injection in management of CVF (52, 53).

### Surgery

We generally consider neurosurgical options in patients who have failed conservative management, not responded to at least two epidural blood patches, and in whom a site of CSF leak has been identified on myelographic imaging that is amenable to surgical treatment. In specific cases, such as CSF venous fistula, surgical option would be considered early in the management algorithm due to ineffectiveness of non-targeted patching in this cohort of patients. Other factors to review when considering surgical management include patient's comorbidities and preferences.

Surgical options utilized are dependent on the underlying etiology but includes ligation of leaking meningeal diverticula and CSF venous fistula, and repair of dural tears. Case control studies from spinal centers specializing in CSF leaks have shown excellent response to surgical treatment with resolution of symptoms in the majority. Hani et al. reported complete resolution of symptoms post-operatively in over 52% of patients and at least partial improvement in 94.2%, although this study did not have any patients with CSF venous fistula (11). A separate study reported very favorable surgical outcomes in patients with CVF with 83% of patients who were in the most severe headache severity category pre-operatively showed major improvement in symptoms (54).

In a study examining predictors of response to surgery for SIH, the preoperative duration of symptoms was the only significant predictor of response, with duration of symptoms under 12 weeks as the cut-off for a better surgical outcome (11). This highlights the need for a timely intervention, which is challenging for numerous reasons, including (1) delays in diagnosis, which may partly be due to the non-specific nature of symptoms associated with SIH, (2) utilization of conservative measures in the first instance once diagnosed, and (3) lengthy waiting times for blood/fibrin patches and myelographic imaging studies, which often also need to be repeated.

### Transvenous embolisation of CSF venous fistula

An emerging less invasive treatment specifically for CVFs is endovascular embolisation of the paraspinal vein draining the CVF. In the largest published case series of 40 patients, treatment was effective in 90% of patients who experience substantial improvement in symptoms, with a similar percentage showing improvement in intracranial MRI features of low pressure on follow-up imaging (median of 3 months). Most common clinical complication was of rebound intracranial hypertension, which was seen in nearly 18% of the patients. Unlike with surgical management of CVF, there was little in the way of post-procedural recovery with all patients in this case series being discharged the same day (55).

## Conclusions and future directions

SIH is a condition with a heterogeneous and evolving clinical presentation. This, together with the variation in clinical practice for investigating SIH, leads to both a delay in, as well as underdiagnosis, of the condition. There is also a lack of uniformity in treatment between different neurosciences units, reflecting the urgent need for consensus guidelines on both the investigation and management of SIH.

Orthostatic headache, the most common symptom of SIH, remains poorly defined with no agreement on the time course of the onset and offset of headache for it to constitute an orthostatic headache, or whether residual mild headache on lying down is permitted. This is vitally important to allow direct comparison of data between studies and thus, facilitate better understanding of the clinical phenotype associated with SIH. The classification of CSF leaks also needs standardizing for the same reasons. Current diagnostic criteria are also in need of refining as seen with the literature surrounding prevalence of low opening pressure in SIH patients.

In the management of SIH, further studies are needed to determine the optimal duration for conservative measures whilst balancing the risk of deconditioning and delaying interventional treatment that can lead to worse clinical outcomes. Studies are also needed to clarify which of the conservative measures lead to the best outcomes. Recent study showing excellent clinical outcomes in patients with CVF in response to percutaneous treatment with targeted fibrin glue occlusion is promising and offers a minimally invasive option in treating CVF. Further studies confirming this finding with longer-term follow-ups are required.

In regard to surgical management, as noted above, early intervention is associated with improved outcomes. Schievink noted 17 out of 18 patients in his study with SIH initially received an incorrect diagnosis with a mean waiting time of 13 months (56). Cheema et al. noted in a UK-based survey that patients with SIH on average saw their general practitioner on three occasions before being referred to a specialist, and even then, in <50% of the patients the correct diagnosis was made by the first specialist they saw. Furthermore, only a third of patients received treatment within 12 weeks of the diagnosis first being considered (8). There needs to be an emphasis on improving awareness of the condition, particularly amongst our general medicine colleagues, to help minimize delay to diagnosis and therefore treatment. Additionally, reducing waiting times for SIH investigations, optimized duration of conservative measures, and early patching in those who require it will allow timely identification of surgical candidates and potentially yield better outcomes.

Finally, more work is needed to determine how best to evaluate and manage the growing number of patients who have clear orthostatic headaches but with normal brain and spinal MRI, particularly when they are not deemed to clinically represent the common differential diagnoses detailed above. We frequently see patients with brain MRI features of intracranial hypotension without spinal abnormalities, and the converse is also true where there is abnormal spinal imaging with normal brain MRI. Thus, it follows that there will be a cohort of SIH patients with normal neuraxial MRI. Neurologists frequently find themselves in a quandary when deciding on whether to offer invasive myelographic testing to this cohort knowing negative tests would not conclusively exclude SIH. Long-term, definitive tests to exclude SIH would help, as would better defining the term “idiopathic” orthostatic headaches. In the interim, consensus on how to best investigate and manage these patients is needed.

## Author contributions

MM and DM were involved in the conception and design of the review. DM wrote-up the draft, whilst contributions in editing and review of the drafts by SC, ID, and MM. ID also helped with acquiring images and labeling of scans for Figure 1. All authors contributed to manuscript revision, read, and approved the submitted version.
